# Effect of Welding Parameters on Al/Mg Dissimilar Friction Stir Lap Welding with and without Ultrasonic Vibration

**DOI:** 10.3390/ma17092109

**Published:** 2024-04-29

**Authors:** Junjie Zhao, Bo Zhao, Chuansong Wu, Najib Ahmad Muhammad

**Affiliations:** 1 School of Materials Science and Engineering, Shandong Jianzhu University, Jinan 250101, China; 2 MOE Key Lab for Liquid-Solid Structure Evolution and Materials Processing, Institute of Materials Joining, Shandong University, Jinan 250061, China; wucs@sdu.edu.cn; 3 Department of Mechanical Engineering, Faculty of Engineering, Bayero University, Kano 3011, Nigeria

**Keywords:** friction stir lap welding, 6061-T6 Al, AZ31B-H24, ultrasonic vibration, welding load, intermetallic compounds

## Abstract

The amount of heat input during welding impacts the weld’s thermal and mechanical behavior and the joint’s properties. The current study involved conducting AA 6061 and AZ31B Mg dissimilar welding, using friction stir lap welding (FSLW) and ultrasonic vibration-enhanced FSLW (UVeFSLW). The comparison and analysis of the welding load, the weld’s macro-microstructure, intermetallic compounds (IMCs), and joint properties were conducted by adjusting the process parameters. The study also examined the effect of ultrasonic vibration (UV) variations on welding heat input. The study demonstrated that it is possible to reduce the welding load by employing UV. Moreover, this impact becomes more pronounced as the welding heat input decreases. Additionally, the material flow in the weld, the width of the weld nugget zone, and the continuous IMC layer are significantly influenced by ultrasonic vibration, irrespective of the heat input during welding. However, the impact on large areas of irregular IMCs or eutectic structures is relatively small. Furthermore, achieving better joint properties becomes more feasible when a higher welding speed is employed for the Al alloy placed on top. Specifically, the impact of UV becomes more evident at higher welding speeds (≥220 mm/min).

## 1. Introduction

The combination of various materials offers distinct benefits in meeting structural performance needs and economic viability, aligning with ongoing industrial production advancements. Al and Mg alloys have been widely used in aerospace, rail transit, shipbuilding, and automotive contexts due to their excellent service performance [1,2]. Using lightweight metals can effectively reduce energy consumption. Conversely, combining Al/Mg materials in hybrid applications can fully exploit their outstanding qualities, expand their application domains, and enhance design adaptability. Nevertheless, when Al/Mg materials are joined, there are unavoidable problems with weldability. These issues occur because Al/Mg materials have a limited capacity to mix, forming numerous hard and brittle IMCs at the Al/Mg interface. This leads to a significant decrease in joint strength [3]. Friction stir welding (FSW) offers distinct advantages for joining dissimilar Al/Mg alloys because of its low heat input during welding [4]. Nevertheless, there is still room for improvement in the properties of Al/Mg FSW joints so that they meet the requirements of industrial production. In essence, the welding parameters should be consistently fine-tuned for maximum efficiency.

IMC generation is affected by the amount of heat input during welding [5,6]. By adjusting the parameters of the welding process, it is possible to effectively modify the heat input, optimize the weld structure, and achieve weld joints of superior quality. For FSLW of dissimilar Al/Mg alloys, the relative position of the base metal, the rotation speed of the tool, the welding speed, and the tool’s shape all impact the weld quality. In earlier studies, researchers employed general welding parameters, which included a tool rotation speed of 400–2800 rpm and a welding speed of 20–150 mm/min [7,8,9,10,11,12,13,14]. Expanding the range of welding process parameters is worthwhile to enhance welding efficiency and improve the quality of the weld joint. 

To prevent the formation of IMCs and enhance the overall quality of the joint, certain researchers have suggested incorporating ultrasonic energy into the FSW process as a supplementary form of energy. Consequently, they have developed multiple techniques for ultrasonic vibration-assisted FSW [15,16,17,18,19]. Several research studies show that ultrasonic vibration can enhance material flow in the weld nugget zone (WNZ), prevent the growth of intermetallic compounds (IMCs), and enhance joint properties. One author’s earlier research also discovered that applying ultrasonic vibration during the Al/Mg FSW process led to a substantial decrease in the thickness of the IMCs on the Al/Mg interface and enhanced the macro-microstructure of the weld in the WNZ [20]. Nevertheless, these comprehensive research studies primarily investigate the influence of ultrasonic vibration on the friction stir welding (FSW) technique using similar or dissimilar materials. The mechanism behind ultrasonic vibration remains to be discovered. Furthermore, the effect of ultrasonic vibration during welding also depends on the material’s temperature (welding heat input) [21,22]. Hence, additional investigation is required to explore the correlation between ultrasonic vibration and welding process variables.

This study aims to optimize welding parameters further and investigate the impact of ultrasonic vibration on various welding parameters. Al and Mg alloys underwent both conventional FSLW and UVeFSLW. In light of the prevalence of lower welding speeds being employed among researchers, this study deliberately widened the range of higher welding speeds. The welding load, weld macro-microstructure, IMCs, and joint tensile strength parameters were obtained. A comparative analysis was conducted to study the effect of welding parameters and the influence of ultrasonic vibration at various levels of welding heat input.

## 2. Experimental Details and Methods

**Figure 1:**
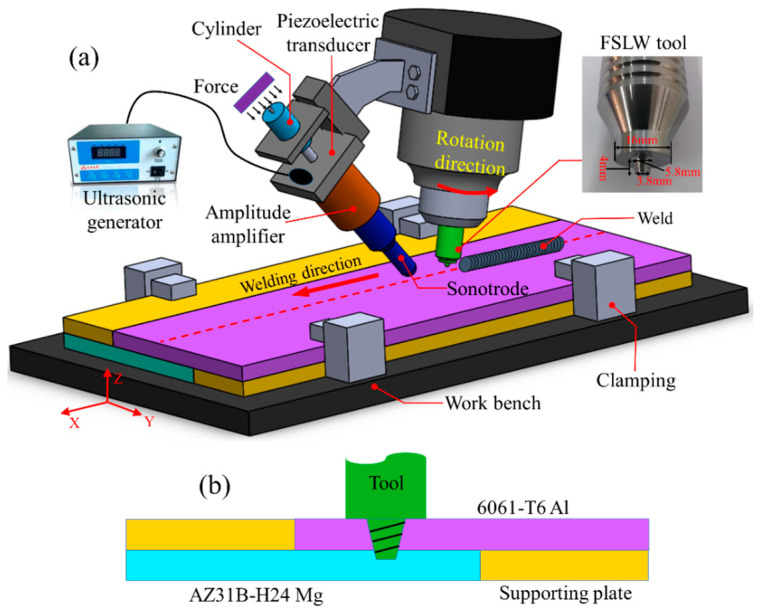
(**a**) Schematics of the Al/Mg FSLW and UVeFSLW process. (**b**) Schematic of the weld configuration.

The experiment used 6061-T6 aluminum alloy and AZ31B-H24 magnesium alloy plates for dissimilar friction stir lap welding. The sizes of the plates were 200 (length) × 100 (width) × 3 (thickness), shown in mm. Their nominal chemical composition is shown in Table 1 [20]. Before welding, the surfaces of the base plates were polished with sandpaper and cleaned with acetone. The welding experiments were conducted using the FSW-3LM-3012 machine (Beijing CFSW Technology Co., Ltd. Beijing, China), and the welding schematic diagram is shown in Figure 1a. At the same time, to further improve the joint’s properties, ultrasonic vibration was used as an auxiliary energy source in the FSLW, i.e., ultrasonic vibration-enhanced friction stir lap welding, or UVeFSLW. As shown in Figure 1a, the sonotrode acted obliquely to the workpiece at a certain angle (set at approximately 40° between the axle of the sonotrode and the surface of the workpieces). In order to achieve the stable transmission of ultrasound energy, the tip of the sonotrode has been designed to be hemispherical. It can move with the tool during the welding process. The ultrasonic action point was located 20 mm in front of the axle of the tool. The working frequency of ultrasonic vibration was 20 kHz, and the output power was about 220 W.

The Al alloy plate was positioned as the upper sheet during welding, as shown in Figure 1b. The overlap width between the Al and Mg alloy plates was 30 mm. Yi et al. [23] placed the workpiece in a water-filled acrylic box and calculated the heat input during the FSW process by measuring the changes in water temperature. They thereby determined a heat input calculation formula, as follows: *HI* ∝ *V*^−0.80^*W*^0.10^*D*^0.55^*d*^0.45^*L*^0.30^λ^0.40^, where HI, V, W, D, d, L, and λ are the heat input (J/mm), welding speed (mm/s), tool rotation speed (rad/s), shoulder diameter (mm), pin diameter (mm), pin length (mm), and thermal conductivity of the workpiece (W/mK), respectively. In the present study, all welding experiments were performed using the same FSW tool. Therefore, the heat input was adjusted by changing the welding and rotation speeds. The welding process parameters were selected as follows: a fixed welding speed of 70 mm/min, the rotation speed of the tool varied from 400–800 rpm, the fixed rotation speed of the tool was 800 rpm, and the welding speed varied from 70 to 460 mm/min. The tool’s inclination angle was 2.5°, and the plunge depth of the shoulder was 0.15 mm. For each process parameter, FSLW and UVeFSLW tests were both conducted. 

The tool with a concave shoulder was made of H13 tool steel. The diameter of the shoulder was 16 mm. The pin was shaped like a frustum with a right-hand thread, and the length, tip diameter, and root diameter were 4 mm, 3.8 mm, and 5.8 mm, respectively.

**Figure 2:**
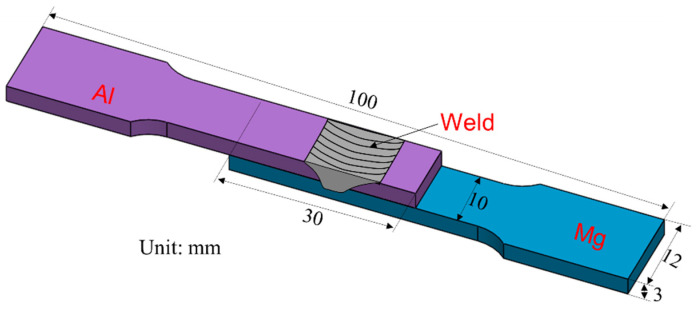
Dimensional details of the tensile shear specimens.

The welding load was recorded during welding experiments to study the effects of welding parameters and UV. After welding, an electric spark wire cutting machine was used to cut the metallographic samples and tensile shear test specimens. The size of the tensile shear test specimens is shown in Figure 2. The tensile shear test was conducted on a CMT-50 universal electronic tensile testing machine with a crosshead speed of 1 mm/min. Each parameter’s joint test was repeated thrice to ensure data reliability.

First, the metallographic samples were ground down and polished. Then, the samples were sequentially etched using NaOH solution (20 g NaOH + 100 mL H_2_O) and picric acid solution (4.2 g picric acid + 70 mL ethanol + 10 mL acetic acid + 10 mL H_2_O). The details of the macro- and microstructures of the weld were obtained with a Zeiss Daheng Invasion USB camera microscope and Keyence Vhx 500F optical microscope. The IMCs generated in the weld were observed and characterized using an SEM (with a JEOL JSM-7800F scanning electron microscope) equipped with an EDS (an energy-dispersive X-ray spectrometer). This equipment was also used to observe the fracture surface morphology of the weld joints.

## 3. Experimental Results and Discussion

### 3.1. Welding Load

**Figure 3:**
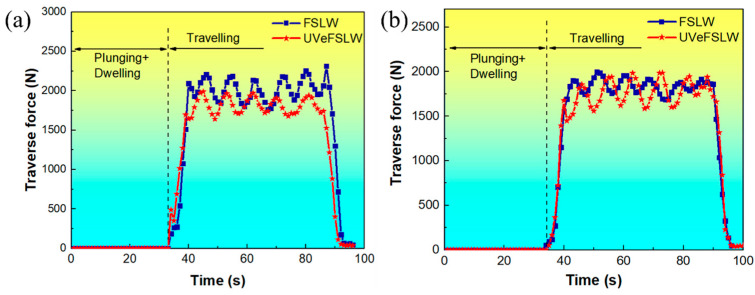
Curves of traverse force in FSLW and UVeFSLW: (**a**) 400 rpm–70 mm/min, (**b**) 800 rpm–70 mm/min.

Welding load is the force that drives material deformation and flow during welding. Large welding loads will not only cause severe wear on the tool but will also lead to the breakage of the pin [24,25]. The larger traverse force is the main reason for this situation. Figure 3 shows the change curves of traverse force under different welding process conditions. The curves have different characteristics at different welding stages. The traverse force is zero since the tool has not moved along the x-axis direction in the plunging and dwelling stages. In the traveling stage, the force increases rapidly and then fluctuates periodically as a sine function. However, some scholars have pointed out that in Al/Mg FSLW, the liquid film generated by constitutional liquation during the welding process can change the friction state between the tool and the material, leading to fluctuations in welding load [7,25]. This sizeable periodic fluctuation could also be related to the screw transmission mechanism of the welding machine itself [24]. 

With the application of UV, the traverse force decreases significantly under a weld parameter of 400 rpm–70 mm/min, as shown in Figure 3a. However, when the welding parameter is 800 rpm–70 mm/min, the UV has a negligible impact on the traverse force, as shown in Figure 3b. This indicates that the reducing effect of UV on traverse force is affected by the welding parameters (welding heat input).

**Figure 4:**
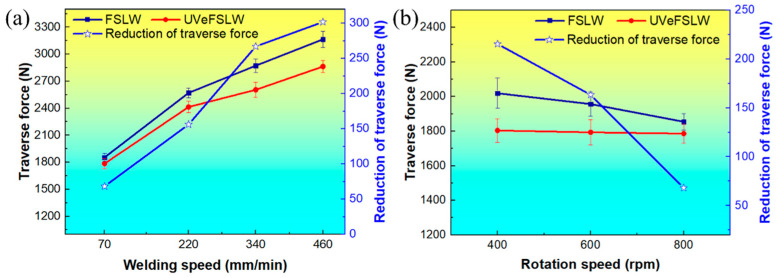
Variations in traverse force under different welding conditions. (**a**) When we fixed the tool rotation speed at 800 rpm, the welding speed varied from 70 to 460 mm/min. (**b**) When we fixed the welding speed at 70 mm/min, the tool rotation speed varied from 400 to 800 rpm.

To better understand the relationship between the effect of UV on traverse force and welding parameters, the change in traverse force under different welding conditions was compared, as shown in Figure 4. The values in the figure are taken from the average value of traverse force in the stable welding stage. With FSLW or UVeFSLW, when the tool’s rotating speed is fixed at 800 rpm, the traverse force increases with the welding speed, as shown in Figure 4a. Moreover, the degree of reduction in the traverse force caused by UV also increases with the increase in welding speed. When the welding speed is at 70 mm/min, the traverse force gradually decreases with the increase in the tool rotation speed in FSLW. However, it remains unchanged with UVeFSLW, as shown in Figure 4b. Generally speaking, the welding heat input is related to the welding parameters. Increasing the welding speed or decreasing the tool rotation speed can reduce the welding heat input [25,26,27]. Therefore, as the welding heat input decreases, the thermal softening degree of the material decreases, and the traverse force increases. The traverse force in FSLW follows this rule, but in UVeFSLW, it has little correlation with the tool rotation speed. This could be related to the effect of UV being influenced by welding heat input.

Applying UV can reduce metal deformation stress by the acoustic softening effect [28,29,30], thus reducing the welding load required during the FSLW process. However, this effect is influenced by the welding temperature. Hung et al. [21] found that the higher the temperature, the smaller the reduction effect of UV on deformation stress during the ultrasonic-assisted hot upsetting process with Al alloys. Zhong et al. [31] also found in FSW that when the welding heat input was more significant, the ultrasonic softening effect was more minor. The reduction of metal deformation stress by UV is mainly achieved by reducing the dislocations’ activation energy, promoting their motion [28]. However, at higher temperatures, the dislocation exhibits high activity, so the effect of UV is weakened at this time.

In addition, compared with changing the rotating speed of the tool, varying the welding speed has a more noticeable effect on the traverse force (Figure 4). This is because the traverse force is mainly the resistance to the forward movement of the tool during welding. When the welding speed increases, the feed rate of the tool increases, and the length of time for the material in front of the tool to soften due to heating is reduced. Therefore, the resistance to material deformation increases.

**Figure 5:**
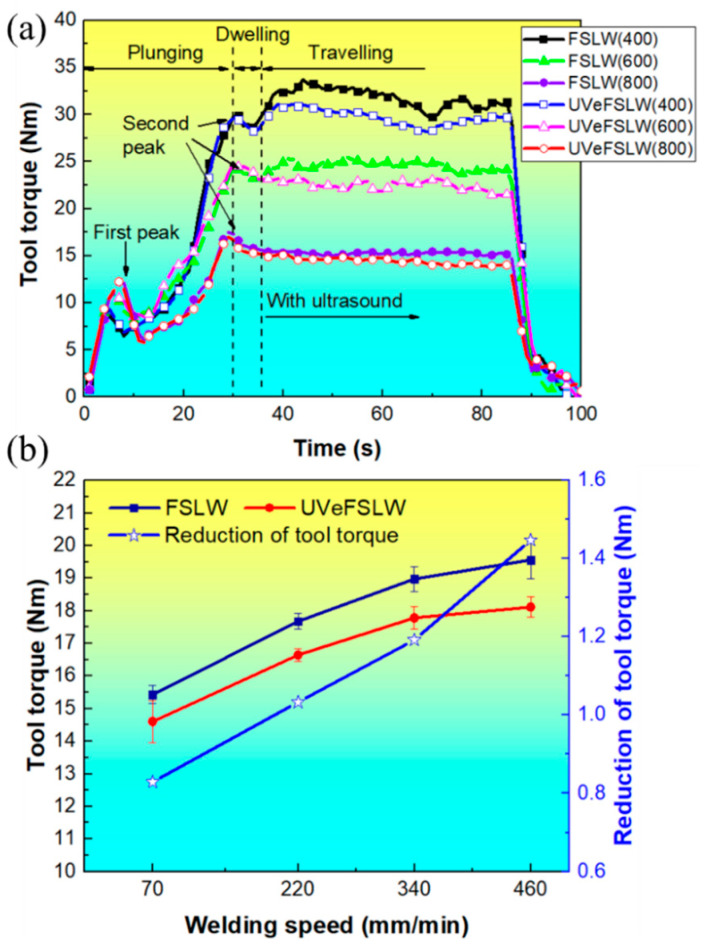
Tool torque under different welding conditions. (**a**) When we fixed the welding speed at 70 mm/min, the tool rotation speed varied from 400 to 800 rpm. (**b**) When we fixed the rotation speed of the tool at 800 rpm, the welding speed varied from 70 to 460 mm/min.

Figure 5 shows the variation in tool torque under different welding process conditions. As the welding heat input decreases (when we reduce the tool rotation speed or increase the welding speed), the tool torque gradually decreases. This is the same as with the change rule of traverse force with welding heat input. However, the tool torque’s curve characteristics differ from those of the traverse force. The tool torque curve has two peaks, as shown in Figure 5a. The first peak occurs during the plunging stage. In this stage, the rotating pin gradually inserts itself into the workpiece. It drives the material toward plastic deformation by the shear and compression effects induced. As the depth of the tool inserting itself into the workpiece increases, the volume of the material that the tool needs to drive increases, and the torque value gradually increases. This situation continues, with the pin plunging in until the frictional heat generated between the pin and the materials can soften the surrounding material. Then, the tool torque value gradually decreases. As the pin is further inserted, the shoulder gradually comes into contact with the materials, and the volume of plastic deformation materials and contact area increases, which causes the tool’s torque to reach the second peak. In the dwelling stage, heat accumulation around the pin can effectively soften the surrounding materials, and the required tool torque also decreases.

When the tool rotation speed is 400 rpm, the torque value increases and decreases slightly in the traveling stage. Meanwhile, it becomes smoother at a higher tool rotation speed, as shown in Figure 5a. Therefore, the higher the welding heat input, the smoother the torque value, which can transition to a stable state. However, during the UVeFSLW process, the transition of torque values to the stable stage is smoother. This is related to the acoustic softening effect and the thermal effect of UV. In addition, the tool torque is also reduced by applying the UV, and the smaller the welding heat input (a lower tool rotation speed), the more significant the acoustic effect (Figure 5). 

**Figure 6:**
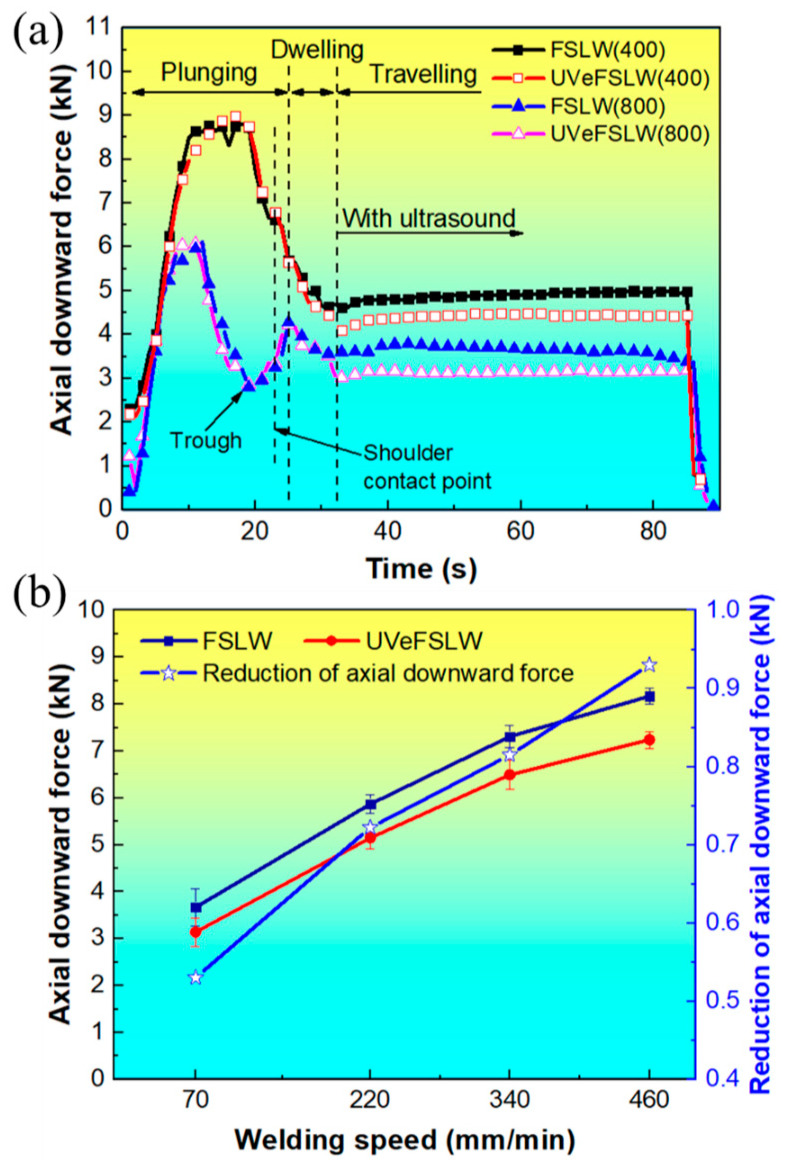
The axial downward force under different welding process conditions. (**a**) When we fixed the welding speed at 70 mm/min, the tool rotation speed varied from 400 to 800 rpm. (**b**) When we fixed the tool rotation speed at 800 rpm, the welding speed varied from 70 to 460 mm/min.

Figure 6 shows the axial downward force under different welding process conditions. In the plunging stage, the curves of the axial downward force rapidly increase at the beginning, reach a brief, stable stage, and then decrease rapidly. As the pin gradually inserts into the workpiece, the friction heat generated between the pin and workpiece and the material’s plastic deformation heat is insufficient to soften the surrounding material fully. Therefore, the required axial downward force increases rapidly until the accumulated heat thoroughly softens the surrounding materials. It then reaches a brief, stable state. As the accumulated heat increases, the required axial downward force also rapidly decreases. 

However, a higher heat input (the tool rotation speed is 800 rpm) can cause this brief, stable stage to arrive earlier and to have a shorter duration. In addition, there is a trough in the axial downward force curve under 800 rpm–70 mm/min. This may be because materials that are easily extruded come into contact with the shoulder earlier at higher heat inputs, increasing the compression area. Therefore, the axial downward force rises again. This also causes the 800 and 400 rpm curves to exhibit different trends at the shoulder contact point. In the dwelling stage, the axial downward force gradually decreases due to the stopping of the tool’s plunging and the continuous heat generation. In the traveling stage, the axial downward force is a stable value that decreases with the application of UV. Moreover, as the welding heat input decreases, that is, as the rotating speed of the tool decreases or the welding speed increases, the effect of UV increases, as shown in Figure 6. This effect is the same as that in traverse force and tool torque.

### 3.2. Macro- and Mesoscopic Structures in Welds

**Figure 7:**
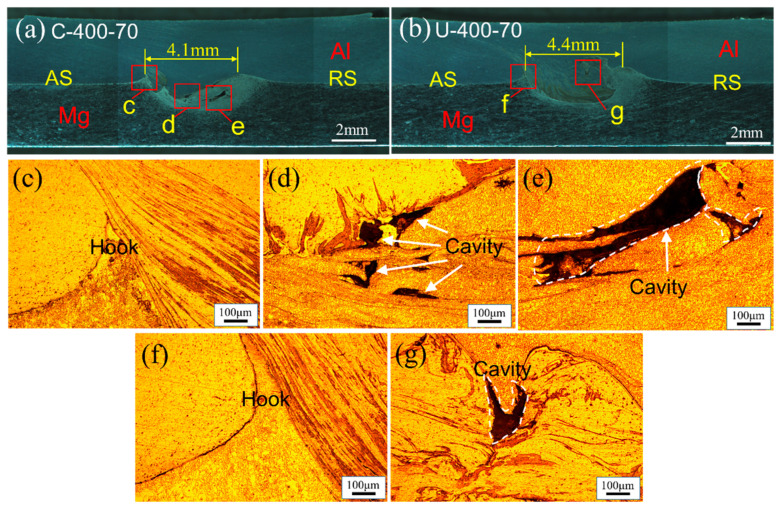
Macro- and mesoscopic structure of welds: (**a**) FSLW-400 rpm–70 mm/min, (**b**) UVeFSLW-400 rpm–70 mm/min, (**c**–**g**) local enlarged images in (**a**,**b**).

Figure 7 shows the macro- and mesoscopic structure images of FSLW and UVeFSLW welds under 400 rpm–70 mm/min. With FSLW, cavity defects of different sizes are observed at multiple locations in the WNZ (Figure 7d,e). This is related to insufficient material flow, which does not effectively fill the space left by the pin feeding, resulting from the lower heat input. The application of UV significantly improved this situation. Figure 7g shows that only a tiny cavity defect was observed. The reason is that UV can reduce the deformation stress of plastic deformation materials, facilitating the material flow in the WNZ [16,21,28,29]. However, the effect of UV on the Hook defect is small, as shown in Figure 7c,f.

In addition, regardless of whether UV is applied or not, there is a significant morphology difference between the AS and RS Hook structures. There is a sharp Mg material protrusion and some slender strips in the Al matrix at the AS (Figure 7c,f). While the strips are absent, the Al/Mg interface curve is relatively smooth at the RS. The reason for this difference is related to the material flow in the WNZ. Moreover, this is a complex flow process that requires further investigation.

**Figure 8:**
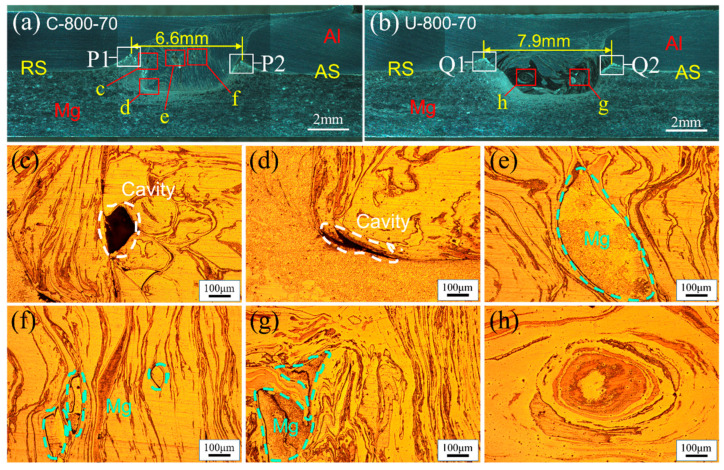
Macro- and mesoscopic structure of welds: (**a**) FSLW-800 rpm-70 mm/min, (**b**) UVeFSLW-800 rpm–70 mm/min, (**c**–**h**) local enlarged images in (**a**,**b**).

Figure 8 shows the macro- and mesoscopic structures of welds under 800 rpm–70 mm/min. As the tool rotation speed increases, the welding heat input increases. However, some cavity defects are still observed with FSLW, as shown in Figure 8c,d. At the same time, some Mg alloy clusters flow into the Al alloy matrix, as shown in Figure 8e,f. With the application of UV, no defects are found in the WNZ, and some Mg alloy fragments or clusters also appear in the Al alloy matrix, as shown in Figure 8g. This indicates that UV can enhance the material fluidity in the WNZ and eliminate welding defects.

The distance between the AS and RS Hook structures is the effective overlap width, which can also represent the volume of the WNZ. The larger the effective overlap width and the longer the interface length, the more conducive this is to the improvement of the shear strength of the joint [32,33]. As shown in Figure 8a,b, with the application of UV, the effective overlap width increased from 6.6 mm to 7.9 mm. Ultrasonic vibration can increase the WNZ volume, contributing to the improvement of joint strength. This effect is consistent with that observed during the ultrasonic-assisted friction stir butt welding of Al/Mg dissimilar alloys [16]. In addition, the flow pattern may be changed due to the material flow being improved by UV. For example, a vortex structure appeared with UVeFSLW, as shown in Figure 8h.

**Figure 9:**
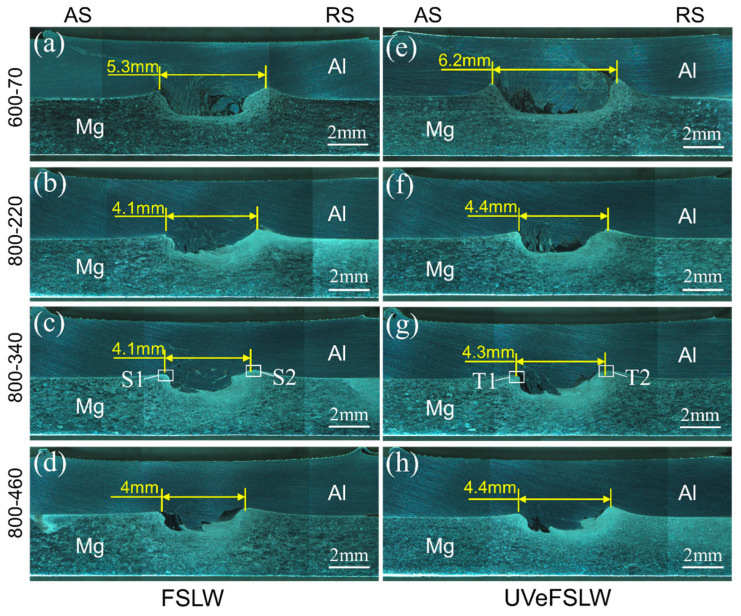
Macro-morphology of FSLW and UVeFSLW welds. (**a**,**e**) 600–70, (**b**,**f**) 800-220, (**c**,**g**) 800–340, (**d**,**h**) 800–460 (rpm-mm/min); (**a**–**d**): FSLW, (**e**–**h**): UVeFSLW.

The macro-morphology of the welds under other welding process conditions is shown in Figure 9. No defects were found in these welds. It is worth noting that when the welding speed is at 70 mm/min, both FSLW welds, made with tool rotation speeds of 400 and 800 rpm, exhibit cavity defects (Figure 7d,e and Figure 8c,d). However, a defect-free weld can be obtained at a rotation speed of 600 rpm (Figure 9a). This is because the lower heat input can cause insufficient material flow, and also, the higher heat input will cause the generation of a large number of IMCs, which both affect the formation quality of the weld [34].

In FSLW, as the tool rotation speed decreases, the width of the WNZ gradually decreases, as shown in Figure 7a, Figure 8a and Figure 9a. As the welding speed increases, the width of the WNZ also decreases (Figure 8a and Figure 9b). However, as the welding speed further increases, the width of the WNZ hardly changes, as shown in Figure 9b–d. The effective overlap width is approximately 4.1 mm in the range of 220 mm/min–460 mm/min, which equals the diameter of the pin. This means that the pin’s driving ability is significantly weakened at higher welding speeds. However, the effective overlap width is approximately 4.4 mm in UVeFSLW welds, as shown in Figure 9f–h. This indicates that UV can significantly increase the volume of deformed material in the WNZ, even at higher welding speeds. We also need to remember that at higher welding speeds, the effect of UV has little correlation with the welding speed. However, it was found that the lower the heat input, the more pronounced the effect of UV on the welding load. Ultrasonic-assisted FSW is a complex coupling process of heat-force-flow-sound. Therefore, the effect of UV on the WNZ width is not simply monotonic, which necessitates further detailed research.

### 3.3. IMCs

For Al/Mg dissimilar FSLW joints, the Hook structures on both sides of the WNZ are essential zones because the stress will first be concentrated here during shear testing [35]. Meanwhile, a large number of IMCs would be generated in these zones, which would seriously affect the strength of the joint. Therefore, a detailed analysis was conducted on these zones.

**Figure 10:**
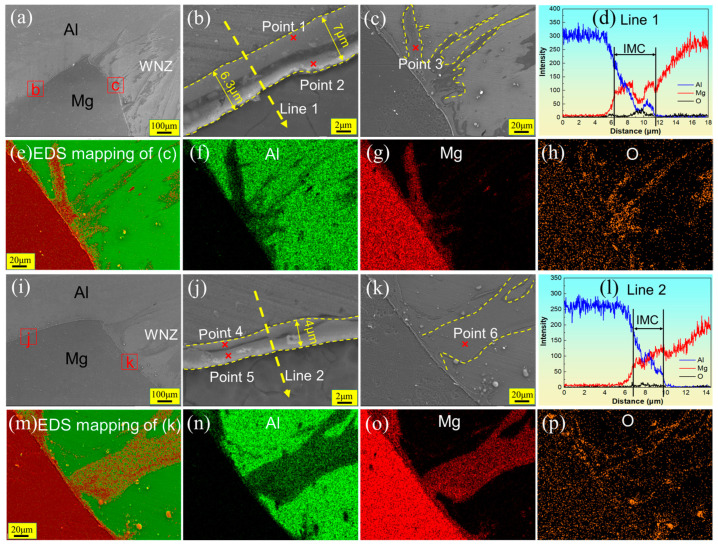
SEM images and EDS results showing Hook structures (corresponding to the white wireframes designated as P1 and Q1 in Figure 8) at the RS in welds under 800 rpm–70 mm/min: (**a**) and (**i**) are Hook structures at the RS with FSLW and UVeFSLW, respectively, (**b**,**c**) and (**j**,**k**) are locally enlarged images, (**d**–**h**,**l**–**p**) are EDS line or map scan results. (**a**–**h**): FSLW, (**i**–**p**): UVeFSLW.

Figure 10 shows the SEM image and EDS results of Hook structures at the RS (corresponding to the white wireframes designated as P1 and Q1 in Figure 8) in the FSLW and UVeFSLW welds. The Hook structure has different morphologies on the left and right sides, as shown in Figure 10a. On the left side, an IMC layer with double sublayers was generated on the Al/Mg interface. As shown in the local enlarged image in Figure 10b, the thickness of the entire IMC layer is about 6.3–7 μm. According to the EDS line scanning (line 1, Figure 10d) and point scanning (points 1 and 2, Table 2) results, the sublayer close to the Al matrix was determined as Al_3_Mg_2_, and the other was Al_12_Mg_17_. It is worth noting that there is a “groove” or “crack” between these two sublayers, indicating that no good metallurgical bonding has formed between them. This is likely to become the crack source or propagation path during the tensile shear test.

Some sputtered structures were found on the right side of the hook structure (near the WNZ), as indicated by the yellow dashed line in Figure 10c. According to the results of EDS mapping (Figure 10e–h) and EDS point scanning (Point 3, Table 2), we can determine that this area shows IMCs, and the type is Al_12_Mg_17_. This large, non-layered structure of IMCs could be generated by constitutional liquation [25]. 

Ultrasonic vibration has little effect on the morphology of the Hook structure at the RS, as shown in Figure 10i, corresponding to the white wireframe marked Q1 in Figure 8. On the left side of the Hook structure, an IMC layer with double sublayers has been generated on the Al/Mg interface (Figure 10j). According to the results of EDS point (Points 4 and 5, Table 2) and line (Line 2, Figure 10l) scanning, the IMC sublayer near the Al matrix comprises Al_3_Mg_2_, while another sublayer comprises Al_12_Mg_17_. The thickness of the entire IMC layer in the UVeFSLW weld is approximately 4 μm, thinner than that with FSLW. In addition, the “groove” or “crack” between these two IMC sublayers is thinner than that with FSLW. This indicates that applying UV is beneficial for improving the metallurgical bonding between materials and reducing the thickness of IMCs.

A sputtered structure was also found on the right side of the Hook structure with UVeFSLW, as shown by the yellow dashed line in Figure 10k. The EDS mapping (Figure 10m–p) results indicate that this area contains many Al and Mg elements. This comprises Al_12_Mg_17_, as proved by the EDS point scanning result (Point 6, Table 2). Moreover, constitutional liquation should also generate the IMCs here [25]. 

**Figure 11:**
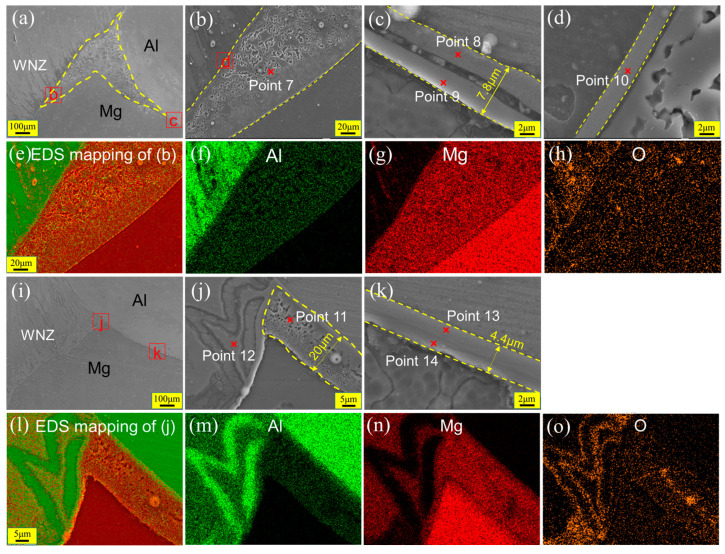
SEM images and EDS results of Hook structures (corresponding to the white wireframes designated as P2 and Q2 in Figure 8) at the AS in welds under 800 rpm–70 mm/min. (**a**,**i**) are Hook structures at the AS with FSLW and UVeFSLW, respectively, (**b**–**d**) and (**j**,**k**) are locally enlarged images, and (**e**–**h**,**l–o**) are EDS map scan results. (**a**–**h**): FSLW, (**i**–**o**): UVeFSLW.

Figure 11 shows the SEM images and EDS results of the Hook structures at the AS with FSLW and UVeFSLW, corresponding to the white wireframes designated as P2 and Q2 in Figure 8. Unlike the situation with the RS, a large number of eutectic structures appear in the upper part of the Hook structure, as indicated by the yellow dashed line in Figure 11a. In the local enlarged image (Figure 11b) and the EDS mapping images (Figure 11e–h) of sub-zone b, the eutectic structure can be observed more clearly. This proves that the temperature on the AS is higher than that on the RS in the Al/Mg friction stir lap welding process. Moreover, it can be confirmed that the eutectic structure is Mg+Al_12_Mg_17_ from the EDS point scanning result (Point 7, Table 2).

It is worth noting that a continuous IMC layer was generated between the eutectic structure and the Al matrix, as shown in Figure 11d. This layer comprises Al_3_Mg_2_, as determined by the EDS point scanning result (Point 10, Table 2). Beygi et al. [36] also found this phenomenon in the FSLW of AA 1050 and AZ91 Mg. They pointed out that this eutectic structure can hinder the growth of continuous IMC layers. However, whether in the case of the IMC layer or the eutectic structure, when its thickness or volume is large, the bonding strength between materials will seriously deteriorate.

On the side farthest from the WNZ, the sub-region c outside the eutectic structure was selected to observe the Al/Mg interface. As shown in Figure 11c, an IMC layer with double sublayers was also generated. Its thickness was approximately 7.8 μm, this being slightly greater than that at the RS (7 μm, Figure 10b). This could be related to the higher welding heat input on the AS. Moreover, there is also a “groove” or “crack” between these two IMC sublayers.

The eutectic structure was also found near the Hook structure at the AS in UVeFSLW, as shown in Figure 11i. It can be observed clearly from the local enlarged image (Figure 11j), as indicated by the yellow dashed line. The maximum width of the eutectic structure is about 20 μm, and the type of IMCs is Mg_12_Al_17,_ as confirmed by the EDS point scanning result (Point 11, Table 2). In addition, from the EDS mapping of subzone j (Figure 11l–o), it can be observed that large-area IMCs also appeared on the left side of the Hook structure. The IMCs are Al_3_Mg_2_, as determined by EDS point scanning (Point 12, Table 2). By comparing the types of large-area IMCs generated in FSLW and UVeFSLW, it can be seen that ultrasonic vibration is more conducive to generating Al_3_Mg_2_. Peng et al. [33] studied the lattice mismatch between different IMCs and Al or Mg matrices and found that their bonding strength, from high to low, is as follows: Al_3_Mg_2_ and Mg, Al_3_Mg_2_ and Al, Al_12_Mg_17_ and Mg, Al_12_Mg_17_ and Al. Therefore, the reduction in eutectic structure with Mg+Al_12_Mg_17_ is significant for improving the joint properties.

On the side nearest the base material zone, an IMC layer was also formed on the Al/Mg interface, as shown in Figure 11k. Interestingly, no obvious “grooves” or “cracks” were found in the IMC layer, which differs from that seen in the FSLW case. However, according to the EDS point scanning results (Points 13 and 14, Table 2), the IMC layer contains two sublayers. The sublayer closest to the Al matrix comprises Al_3_Mg_2_, while another comprises Al_12_Mg_17_. The thickness of the IMC layer is 4.4 μm, which is smaller than that in FSLW. Therefore, these phenomena prove that, once again, the application of UV can suppress the growth of IMCs.

**Figure 12:**
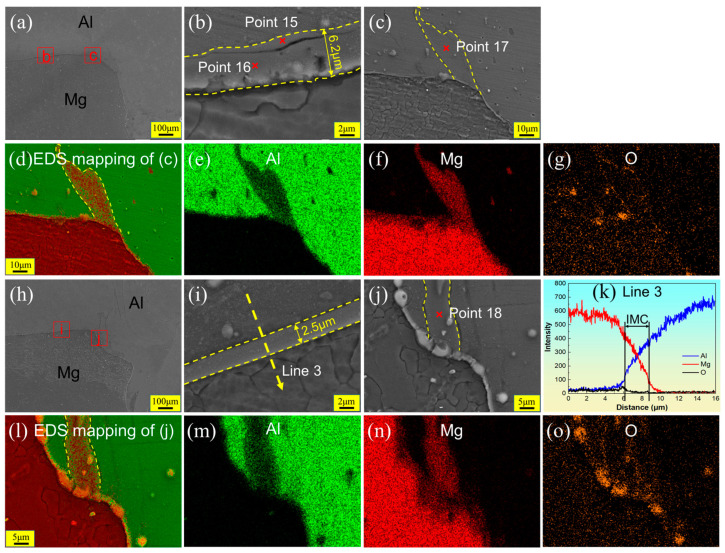
SEM images and EDS results of Hook structures (corresponding to the white wireframes designated as S1 and T1 in Figure 9) at the AS in welds under 800 rpm–340 mm/min: (**a**,**h**) are Hook structures at the AS in FSLW and UVeFSLW, respectively, (**b**,**c**) and (**i**,**j**) are locally enlarged images, and (**d**–**g**,**k**–**o**) are EDS line/map scan results. (**a**–**g**): FSLW, (**h**–**o**): UVeFSLW.

The generation of IMCs is related to welding heat input. When using a lower heat input, the generation of IMCs can be significantly suppressed [5]. Figure 12 shows the SEM images and EDS results of Hook structures at the AS in the FSLW and UVeFSLW samples with welding parameters of 800 rpm–340 mm/min, corresponding to the white wireframes designated as S1 and T1 in Figure 9c,g. Unlike with a welding speed of 70 mm/min, no large area of eutectic structure is observed here. A continuous IMC layer formed on the Al/Mg interface, as shown in Figure 12b. The IMC layer also contains two sublayers with a thickness of approximately 6.2 μm. A “groove” or “crack” exists between the two sublayers. The sublayer close to the Al matrix comprises Al_3_Mg_2_, and the other comprises Al_12_Mg_17_. And the thickness of the Al_12_Mg_17_ sublayer is significantly greater than that of Al_3_Mg_2_. Some scholars have pointed out that Al_12_Mg_17_ will first be generated on the Mg side, due to its lower eutectic temperature [34,35]. However, in general, Al_3_Mg_2_ has a faster growth rate [34,37]. This phenomenon may be because the lower welding heat input is not conducive to the growth of Al_3_Mg_2_, but further research is needed.

As shown in Figure 12c–g, a large area of irregularly shaped IMCs was also found in the local area near the Al/Mg interface. According to the EDS point scan results (Point 17, Table 2), it is confirmed that its component is Al_3_Mg_2_. The IMCs could be generated by constitutional liquation [25], indicating that local liquation also occurs in Al/Mg lap welds with higher welding speeds. However, this is rarely observed in Al/Mg butt welding, especially at lower heat inputs [20,38].

No eutectic structure was found near the Hook structure in UVeFSLW, as shown in Figure 12h. Figure 12i, taken from the enlarged local image, shows a continuous IMC layer formed on the Al/Mg interface. Its thickness is about 2.5 mm, which is significantly lower than that in FSLW (6.2 μm). IMCs comprise a double sublayer structure, as confirmed by EDS line scanning (Line 3, Figure 12k). However, no “cracks” or “grooves” were found in the IMCs. This also indicates that the excitation of ultrasonic vibration can reduce the thickness of IMCs and promote metallurgical bonding between materials.

From the SEM image (Figure 12j) and the EDS mapping (Figure 12l–o), a large area of IMCs was also identified near the Al/Mg interface in UVeFSLW; its composition can be confirmed as Al_12_Mg_17_. Although large areas of IMCs were generated near the Hook structure at the AS in both FSLW and UVeFSLW, their influence on the bonding strength of Al/Mg materials is weaker than that of the continuous IMC layer formed on the interface. Therefore, it is evident that ultrasonic vibration reduces the thickness of continuous IMCs and eliminates “grooves” or “cracks” between the IMC sublayers, which improves joint strength.

**Figure 13:**
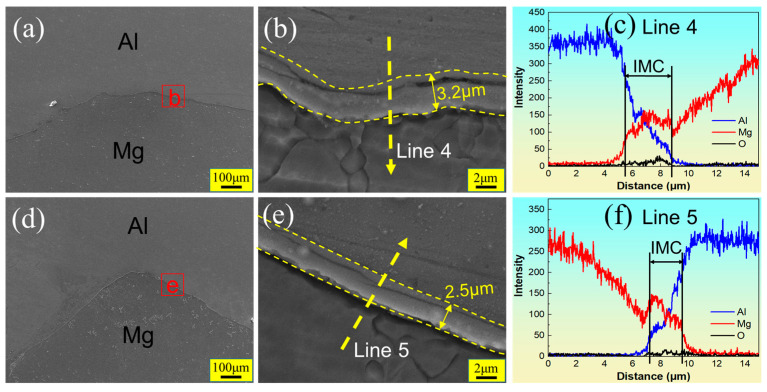
SEM images and EDS results of the Hook structures (corresponding to the white wireframes designated as S2 and T2 in Figure 9) at the RS in welds under 800 rpm–340 mm/min. (**a**,**d**) are Hook structures at the RS in FSLW and UVeFSLW, respectively; (**b**,**e**) are locally enlarged images, and (**c**,**f**) are EDS line scan results. (**a**–c): FSLW, (**d**–**f**): UVeFSLW.

Figure 13 shows the SEM images and EDS results of the Hook structure at the RS in FSLW and UVeFSLW, corresponding to the white wireframes designated as S2 and T2 in Figure 9c,g. No large-area eutectic structure or IMCs were found near the Hook structure, as shown in Figure 13a,d. This could be because, at lower heat inputs, the temperature on the RS is too low to cause local liquefaction. The continuous IMC layer is still generated on the Al/Mg interface (Figure 13b,e). In FSLW, the thickness of the IMCs is approximately 3.2 μm, which significantly lowers the case of the welding speed to 70 mm/min (6.3–7 μm, Figure 10b). According to the EDS line scanning result (Line 4, Figure 13c), these IMCs have double sublayers and a “crack” or “groove” between them. After applying ultrasonic vibration, an IMC layer with double sublayers was also formed on the interface, as confirmed by the EDS line scanning result (Line 5, Figure 13f). However, the thickness of the IMCs decreased to 2.5 μm, and the crack between the two sublayers became smaller.

Comparing the IMCs generated near the Hook structure at 70 and 340 mm/min welding speeds, regardless of the welding heat input, a continuous IMC layer will always be generated on the Al/Mg interface. Moreover, these IMC layers all have a double sublayer structure. It is worth noting that the IMCs in FSLW generally have a “groove” or “crack” between their two sublayers. The excitation of ultrasonic vibration can significantly improve this situation and reduce the entire thickness of IMCs. During the welding process, the temperature of the material near the Hook structure is lower, and it is difficult to form a mechanically mixed structure between Al and Mg with a lower degree of deformation.

Meanwhile, some impurities or oxides remaining on the welding surface can also affect the metallurgical bonding between materials. Therefore, “grooves” or “cracks” are prone to occur between the double sublayers of IMCs. The excitation of UV can significantly improve the material flow in the WNZ and, thus, contribute to the material flow near the hook structure. At the same time, high-frequency vibrations also contribute to the fragmentation of residual oxide films. Therefore, “grooves” or “cracks” can be significantly eliminated and alleviated with UVeFSLW.

### 3.4. Tensile Shear Strength of Joints

**Figure 14:**
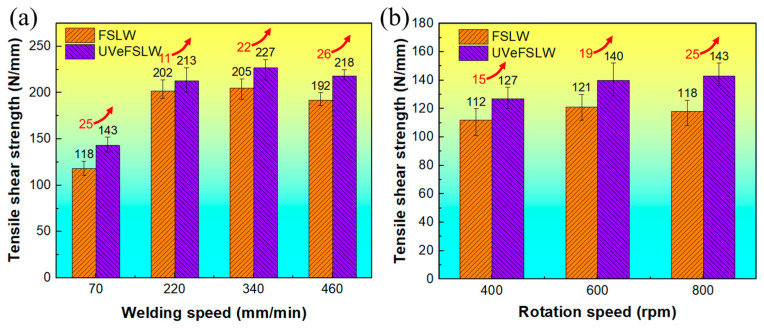
Tensile shear strength of FSLW and UVeFSLW joints. (**a**) When we fixed the tool rotation speed at 800 rpm, the welding speed varied from 70 to 460 mm/min. (**b**) When we fixed the welding speed at 70 mm/min, the tool rotation speed varied from 400 to 800 rpm.

Figure 14 shows the tensile shear strength of FSLW and UVeFSLW joints under different welding process conditions. In FSLW, when the tool rotation speed is fixed at 800 rpm and with the welding speed increasing, the joint tensile shear strength first increases and then decreases (Figure 14a). The maximum tensile shear strength is obtained at a 340 mm/min welding speed. When the welding speed is fixed at 70 mm/min, the tensile shear strength of the joint does not change greatly with changes in the tool rotation speed, as shown in Figure 14b. The lowest tensile shear strength is obtained at 400 rpm, which may cause many defects in the weld. Comparing Figure 14a,b, the higher tensile shear strength is more easily obtained by changing the welding speed. The tensile shear strength at higher welding speeds (more than 220 mm/min) is about 80 N/mm higher than that at lower welding speeds. This indicates that increasing the welding speed is a feasible method to obtain high-strength Al/Mg dissimilar FSLW joints. This is because the heat input is lower at higher welding speeds, and the number and volume of the IMCs generated in the weld, especially near the Hook structure, are significantly reduced. At the same time, this is also beneficial for improving welding efficiency.

With the application of UV, the tensile shear strength under all welding parameters improves. Moreover, the highest strength with UVeFSLW is still obtained at 800 rpm–340 mm/min, reaching 227 N/mm. It is worth noting that when the tool rotation speed is fixed at 800 rpm, with the welding speed increasing, the improvement value of joint tensile shear strength by the excitation of UV first decreases and then increases, as shown by the red numbers in Figure 14a. However, the effect of UV on joint tensile shear strength increases with a higher welding speed (above 220 mm/min). This is in line with the previous action law of UV regarding welding load. That is, the lower the welding heat input, the more obvious the effect of UV. However, when the welding speed is fixed at 70 mm/min, as the tool rotation speed increases (the heat input increases), the effect of UV becomes more apparent, as shown by the red numbers in Figure 14b. This contradicts the law of UV action when changing the welding speed. For Al/Mg dissimilar FSLW, many factors, such as IMCs, mechanical mixing between materials, welding defects, etc., affect the joint properties [32,35,38].

Moreover, these factors do not vary monotonically with the increase in welding heat input. This complex coupling process requires further research to clarify the relationship between the ultrasonic energy field and the welding thermal-force-flow field. However, applying UV can undoubtedly promote material flow in the WNZ, reduce the generation of welding defects, and reduce the thickness of the IMC layer [15,16,20]. Therefore, UV can be a feasible way to improve the strength of Al/Mg FSLW joints. 

**Figure 15:**
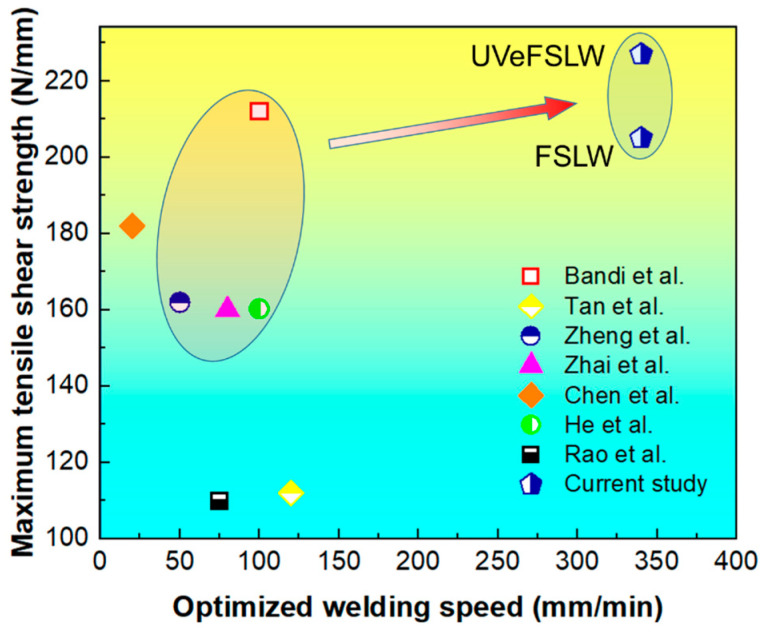
Comparison of the findings on joint tensile shear strength versus optimized welding speed with the results obtained from the literature [8,9,12,13,32,39].

For the FSLW of dissimilar Al alloy and Mg alloy, adjusting the relative position of the base metals, the welding speed, and the tool rotation speed are all effective ways to improve the joint properties. This study found that increasing the welding speed can increase joint strength more easily when placing the Al plate on the top. Moreover, the results of this study are significantly better than those of many researchers, as shown in Figure 15. This validates the possibility of achieving a highly efficient and high-quality Al/Mg FSLW joint by using a higher welding speed. In addition, the application of UV can further improve the joint strength.

### 3.5. Analysis of the Joint Fracture Location and the Fracture Surface

**Figure 16:**
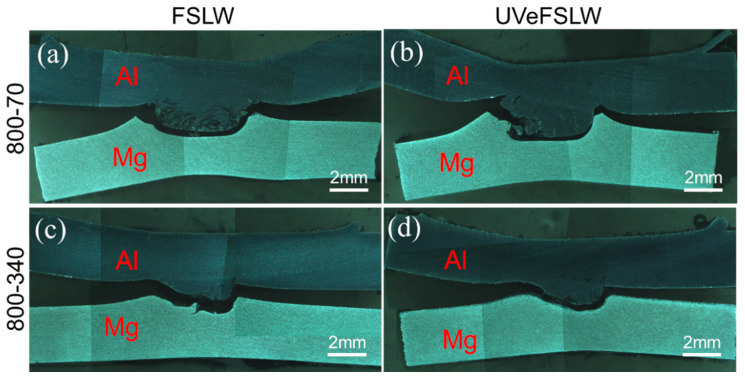
Images of joint fracture locations: (**a**,**b**) 800 rpm–70 mm/min, (**c**,**d**) 800 rpm–340 mm/min. (**a**,**c**): FSLW, (**b**,**d**): UVeFSLW.

In this study, regardless of whether or not UV was applied, all joints fractured along the Al/Mg interface. The fracture location images of joints under 800 rpm–340 mm/min (lower welding input) and 800 rpm–70 mm/min (higher welding input) were selected for display, as shown in Figure 16. There are two reasons for this phenomenon: (1) there is no mechanical interlocking between Al/Mg materials; (2) a large number of IMCs are generated on the Al/Mg interface.

**Figure 17:**
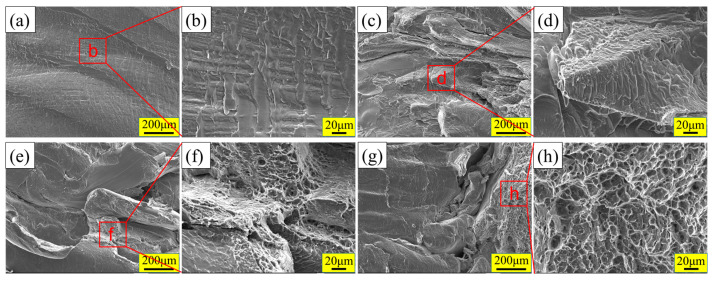
SEM images of the fracture surface: (**a**–**d**) 800 rpm–70 mm/min, (**e**–**h**) 800 rpm–340 mm/min; (**a**,**b**,**e**,**f**): FSLW, (**c**,**d**,**g**,**h**): UVeFSLW.

Figure 17 shows SEM images of the joint fracture surfaces. When the welding speed is 70 mm/min, the fracture surface is relatively flat in the FSLW joint. The scaly structure can be observed from the local enlarged image (Figure 17a,b), which belongs to a brittle fracture. With the application of UV, tearing edges can be found on the fracture surface, but these still belong to the brittle fracture (Figure 17c,d). When the welding speed is 340 mm/min (the joint with the highest tensile shear strength), although some dimples can be observed on the fracture surface for FSLW and UVeFSLW joints, their area is small. Therefore, both the FSLW and UVeFSLW joints still belong to brittle fractures.

## 4. Conclusions

(1) When different heat inputs are used in welding, this results in different curve characteristics in the welding load, particularly with the axial downward force during the plunging stage. By exerting UV, the welding load can be substantially reduced. This encompasses the traverse force, tool torque, and axial downward force. The effect of UV becomes more apparent as the heat input decreases.
(2) With Al/Mg dissimilar FSLW, too high (800 rpm–70 mm/min) or too low (400 rpm–70 mm/min) a welding heat input will lead to welding defects. Applying UV can promote the material flow, reduce, or even eliminate the welding defects, and increase the WNZ volume. Moreover, the correlation between the effect of UV on the WNZ volume and the welding heat input is relatively tiny at higher welding speeds.
(3) A continuous IMC layer with a double sublayer structure is always generated on the Al/Mg interface, whether the welding heat input is high (800 rpm–70 mm/min) or low (800 rpm–340 mm/min). Moreover, with FSLW, grooves or cracks between the two sublayers are present. Applying UV can reduce the thickness of entire IMC layers, narrow down, or eliminate the grooves or cracks between sublayers.
(4) Large areas of irregularly shaped IMCs or eutectic structures can occur near the Hook structure, especially on the AS. Regardless of a high or low welding heat input, ultrasonic vibration cannot eliminate their generation, and the effect is also not noticeable.
(5) It is easier to obtain greater joint strength by increasing the welding speed than by changing the tool rotation speed. This study obtained the highest joint strength for FSLW and UVeFSLW at 800 rpm–340 mm/min.
(6) Ultrasonic vibration can improve joint strength at various process parameters. When the welding speed is 70 mm/min, the influence of UV becomes more significant with increasing tool rotation speeds. The impact of UV becomes more apparent as the welding speed increases to 220 mm/min or higher.

## Figures and Tables

**Table 1:** Chemical composition (wt%) and mechanical properties of the base materials.

Alloy	Si	Fe	Cu	Mn	Mg	Cr	Zn	Al	UTS(MPa)	EL (%)
6061-T6	0.51	0.20	0.30	0.009	1.09	0.13	0.05	Bal.	294	10
AZ31B-H24	0.016	0.001	0.003	0.44	Bal.	-	1.10	3.05	260	12

**Table 2:** EDS scanning results at each point in Figure 10, Figure 11 and Figure 12 and the possible phases.

Points	Chemical Composition (at%)			Reference Phase
	Al	Mg	O	
1	55.5	37.0	7.5	Al_3_Mg_2_
2	34.4	53.2	12.4	Al_12_Mg_17_
3	38.4	56.0	5.7	Al_12_Mg_17_
4	59.8	31.5	8.7	Al_3_Mg_2_
5	41.6	50.9	7.5	Al_12_Mg_17_
6	39.0	54.3	6.7	Al_12_Mg_17_
7	35.1	58.3	6.5	Mg+Al_12_Mg_17_
8	57.1	34.9	8.0	Al_3_Mg_2_
9	59.3	36.7	4.0	Al_3_Mg_2_
10	58.5	32.0	9.5	Al_3_Mg_2_
11	31.6	55.9	12.5	Al_12_Mg_17_
12	58.5	36.2	5.4	Al_3_Mg_2_
13	60.6	35.8	3.6	Al_3_Mg_2_
14	39.5	51.7	8.8	Al_12_Mg_17_
15	33.6	56.3	10.1	Al_12_Mg_17_
16	47.8	33.1	19.1	Al_3_Mg_2_
17	56.3	37.4	6.4	Al_3_Mg_2_
18	42.7	51.6	5.7	Al_12_Mg_17_

## Data Availability

Data are contained within the article.
